# Inorganic and Organic Hybrid Nanoparticles as Multifunctional Crosslinkers for Rubber Vulcanization with High-Filler Rubber Interaction

**DOI:** 10.3390/polym10101138

**Published:** 2018-10-12

**Authors:** Lijuan Chen, Xiaohui Guo, Yuanfang Luo, Zhixin Jia, Yongjun Chen, Demin Jia

**Affiliations:** 1 Key Lab of Guangdong High Property and Functional Macromolecular Materials, Department of Polymer Materials and Engineering, South China University of Technology, Guangzhou 510640, China; scutchenlj@163.com (L.C.); 15602325346@163.com (X.G.); yjchen@scut.edu.cn (Y.C.); psdmjia@scut.edu.cn (D.J.); 2 Guangdong Provincial Key Laboratory of Functional Soft Condensed Matter, Department of Polymeric Material and Engineering, School of Materials and Energy, Guangdong University of Technology, Guangzhou 510006, China

**Keywords:** organic and inorganic hybrid, rubber vulcanizator, functional nanoparticle, interfacial interaction, mechanical properties

## Abstract

Improving the interfacial interaction between rubber and silica nanoparticles, and simultaneously reducing free sulfur and preventing migration and volatilization of a rubber vulcanizing agent, commercial sulfur compound aliphatic ether polysulfide (VA-7) was chemically attached to the silica surface to obtain a functionalized nanoparticle (silica-s-VA7). Functional nanoparticles can not only effectively crosslink rubber without sulfur as a novel vulcanizator, but are also evenly dispersed in the rubber matrix and improve the dispersion of the remaining pristine silica as an interfacial compatibilizer. In addition, the thicker immobilized polymer layer and prominent crosslinking density of SBR nanocomposites simultaneously demonstrate that the novel vulcanizing agent silica-s-VA7 gives rise to significant improvement on the rubber–filler interfacial adhesion on account of the covalent linkages of organic and inorganic interfaces between elastomer and nanofillers. We envisage that this strategy may provide a new avenue to implement high-efficiency design for a multifunctional rubber-vulcanizing agent through an organic and inorganic hybridization mechanism.

## 1. Introduction

Rubber is a necessary material for the manufacture of military products, automobiles, aerospace-related products, medical products, and daily necessities [1]. Depending on its source, rubber can be divided into natural and synthetic rubber. The preparation of synthetic rubbers developed rapidly since the early 20th century. Compared with natural rubber, the performance of synthetic rubber is weaker due to a lower molecular weight and shorter molecular chain [2,3]. However, due to the influence of polar groups on synthetic-rubber molecular chains, synthetic rubber possesses special properties such as insulation, airtightness, oil resistance, and low- and high-temperature resistance [4,5]. Meanwhile, styrene butadiene rubber (SBR) is an important synthetic rubber, especially in the rubber-tire industry.

Aliphatic ether polysulfide (VA-7) is a special sulfur donor with special uses in the rubber industry. It is a sticky yellow liquid, and the molecular structure of VA-7 is similar to that of bis [3-(triethoxysilyl)propyl] tetrasulfide (TESPT) silane coupling agent with tetrasulfide (–S–S–S–S–) in the molecular chain. VA-7 can be used in natural rubber and various synthetic rubbers as a kind of vulcanizing agent, or used in combination with other vulcanizing agents as an assistant vulcanizing agent. Because of its special long-chain structure, it can uniformly disperse in the rubber matrix with remarkable filler-rubber interfacial interaction. Because of the complex mechanism of the curing process, the curing procedures of the VA-7 vulcanization system have followed established curing procedures such as initiation, induction, activation, and sulfurization, yet crosslinking [6,7] especially works as the TESPT vulcanization system due to a similar molecular structure [8]. The vulcanizate cured by VA-7 gives priority to produce mono- and disulfide crosslinks with higher crosslinking efficiency, and has better thermal stability than polysulfide crosslinks. Therefore, the vulcanizate cured by VA-7 can obtain pre-eminent mechanical properties such as excellent thermal-oxidative aging, higher tensile strength, and smaller deformation. Since VA-7 has a special aliphatic ether bond structure, it does not generate free sulfur in the vulcanizates, with no copper corrosion, and has been widely used in the field of rubber-sheathed wires and cables instead of insoluble sulfur. Furthermore, vulcanizing agent VA-7 does not produce toxic and harmful substances during rubber vulcanization and does not have the phenomenon of volatilization and migration to product surface, giving rise to environmental pollution. Hence, VA-7 is a green vulcanizing agent in the rubber industry.

Recently, our team has successfully prepared several filler-supported rubber additives via the chemical reaction between the filler and rubber additives with a specific active group [9,10,11,12]. The filler-supported additives can remarkably increase aging resistance and vulcanizing or accelerating efficiency compared to that of the traditional additives, while simultaneously improving the physical mechanical properties of nanocomposites, such as excellent filler–rubber interfacial interaction, superior crosslinking density and tensile strength, thicker immobilized polymer layer, lower rolling resistance, and outstanding wet resistance. These filler-supported rubber additives can also overcome the weakness of traditional small molecular additives such as high volatility and migration due to the lower solubility.

In this article, we have prepared a novel organic–inorganic hybrid vulcanizator (silica-s-VA7) with VA-7 as the organic phase and silica particles as the inorganic phase via the hydrogen bond interaction between silanol hydroxyl and the ether bond on the VA-7 molecular chains. Meanwhile, silica-s-VA7 can effectively vulcanize SBR to form a crosslinking network instead of sulfur. For this organic-inorganic hybrid functional nanoparticle, it is not only a novel vulcanizator, but also a modified filler to form the crosslinking network between rubber molecular chains and filler, simultaneously improving filler dispersion in the rubber matrix. The effects of silica-s-VA7 on curing properties, filler dispersion, filler-rubber interfacial interaction, mechanical properties, and crosslinking density were comprehensively investigated. The desirable organic-inorganic hybrid vulcanizator silica-s-VA7 is better than insoluble sulfur due to its significant advantages of high performance, low volatility and mobility, and environmental friendliness, being a good candidate for the preparation of functional nanofillers in the rubber industry.

## 2. Experimental Section

### 2.1. Materials

Styrene–butadiene rubber SBR1502 (SBR) was purchased from the Guangzhou Institute of Rubber Products, China. Precipitated silica (FINE-SIL518) with a median particle with 5 µm diameter and specific surface of 200–220 m^2^/g was produced by the Jiangxi Huiming Chemical Co., Ltd., Yichun, China. Aliphatic ether polysulfide (VA-7) was obtained from the Jiangsu China Star New Materials Technology Co., Ltd., Suqian, China. Tetrasulfide (TESPT) was purchased from Alfa Aesar, Shanghai, China. Accelerator n-cyclohexyl-2-benzothiazolesulfenamide (CBS), antioxidant n-1,3-dimethylbutyl-N′-phenyl-p-phenylenediamine (DMPPD), activator zinc oxide (ZnO), stearic acid (SA) and insoluble sulfur (S) were industrial-grade products obtained from the Zhejiang Huangyan Zhedong Rubber Chemicals Co., Ltd., Huangyan, China, and used as received. Petroleum ether, acetone and toluene were analytical reagents purchased from Damao Chemical Reagent Factory, Tianjin, China, and used as received.

### 2.2. Preparation of Silica-s-VA7 Vulcanizator

**Figure 1:**
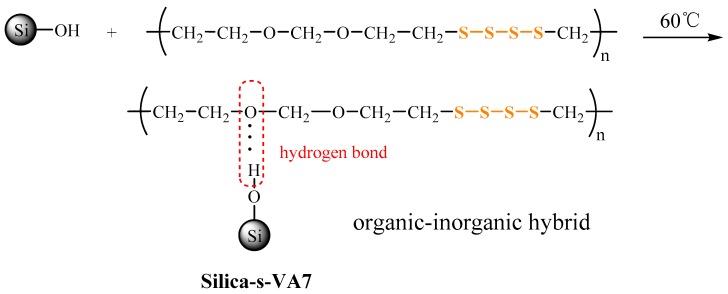
Synthetic route of silica-s-VA7.

The silica-s-VA7 was prepared by the physical interaction of hydrogen bonding between the silanol groups on the silica surface and the ether bond on the VA-7 molecular chain. The synthetic route of silica-s-VA7 is shown in Figure 1. Firstly, 10, 20, and 30 g of silica were ultrasonically dispersed in 300 mL of petroleum ether, respectively, and then 10 g VA-7 was dissolved in a certain amount of toluene and dropwisely added into the suspension. The mixture was stirred for 8 h at 60 °C in 3-necked flask equipped with a reflux condenser under a nitrogen atmosphere. The product was filtered and washed with 200 mL of toluene and 200 mL of petroleum ether, each 3 times. The modified silica (SV1 stands for 10 g silica vs. 10 g VA-7, SV2 stands for 20 g silica vs. 10 g VA-7, and SV3 stands for 30 g silica vs. 10 g VA-7) was dried in vacuum-oven to a constant weight at 80 °C.

### 2.3. Preparation of SBR/Silica Nanocomposites

SBR/silica nanocomposites vulcanized by different vulcanizing agents were prepared according to the formulas in Table 1. The constant filler content for each sample was 38 phr (total filler content for SBR/silica-s-VA7 composites included neat silica and the calculated carrier silica of SV1, SV2, and SV3, respectively), and the sulfur content for each sample was approximately 2 phr. The mixing of components was implemented in an open 2-roll mill for 10 min. After homogenization, composites were further molded at 160 °C for the optimum cure time (T_90_).

### 2.4. Characterization

Fourier transform infrared (FTIR) measurements were carried out on a Bruker Vector 33 spectrometer (Bruker Corporation, Billerica, MA, USA) in the range of 400–4000 cm^−1^. Raman spectra were determined using an HJY Aramis microprobe Raman spectroscopy (HORIBA Jobin Yvon, Ltd., Paris, France) in the range of 100–4000 cm^−1^ at a wavelength of 523 nm. Thermogravimetric analysis (TGA) was conducted under a nitrogen atmosphere with a TA Q5000 (TA Instruments, Inc., New Castle, PA, USA) at a heating rate of 20 °C/min from 30 to 700 °C. Experimental loading amount *C*_A_ [13] of VA-7 was determined by the weight loss from the TGA and calculated with Equation (1):

(1) $$C_{A}=\left(1-w_{\mathrm{silica-s-VA}7}/w_{\mathrm{silica}}\right)\times 100\%$$

where $C_{A}$ is the experimental loading amount of VA-7; $w_{\mathrm{silica-s-VA}7}$ is the weight of VA-7-loaded silica at 700 °C; and $w_{\mathrm{silica}}$ is the weight of silica at 700 °C. The sulfur content of each silica-supported VA7 vulcanizators was carried out on a Vario EL cube ELEMENTAR elemental analyzer (Elementar Analysensysteme GmbH, Langenselbold, Germany). The vulcanization characteristics of SBR composites were determined by rotorless rheometer UR-2030 (U-CAN DYNATEX Inc., Taichung, Taiwan) at 160 °C. Scanning electron microscopy (SEM) was performed on a ZEISS Merlin (Carl Zeiss, Jena, Germany) at an acceleration voltage of 10.0 kV, and samples (dumbbell shape: 20 mm × 6 mm × 1 mm) were fractured after fast cooling by immersing in liquid nitrogen. Fracture surface was sprayed with gold before testing. Strain sweeps of unvulcanized and vulcanized compounds were measured on a Rubber Processing Analyzer (α, RPA 2000, 03B1 Technologies, Hudson, OH, USA) from 0.7% to 200% at 60 °C, 1 Hz. Tensile and tear tests were performed on a U-CAN UT-2060 instrument according to ASTM D 412 (dumbbell A shape) and ASTM D 624 (nicked angle) (U-CAN DYNATEX Inc., Taiwan), respectively, at a crosshead speed of 500 mm/min. The thickness of the samples was 1.0 mm. Crosslinking density was determined by equilibrium-swelling method as previously reported [10,13], and the vulcanized SBR composites, with a different vulcanizing agent, were made into the required shape (30 mm × 5 mm × 2 mm).

Heat-capacity curves were obtained using a NETZSCH DSC 204 F (NETZSCH-Gerätebau GmbH, Selb, Germany). Each sample was first isothermal for 5 min at −100 °C; this was followed by heating at a rate of 10 °C/min to 30 °C under a nitrogen atmosphere. The experimental parameters of $\Delta C_{pn}$ and weight fraction of immobilized polymer layer *χ_im_* were assigned to the heat-capacity step as per the literature [14,15,16,17,18]. $\Delta C_{pn}$ and $\chi_{im}$ were calculated as Equations (2) and (3):

(2) $$\Delta C_{pn}=\Delta C_{p}/(1-w)$$

(3) $$\chi_{im}=(\Delta C_{p0}-\Delta C_{pn})/\Delta C_{p0}$$

$\Delta C_{p}$ is the heat-capacity jump at T_g_ of the SBR/silica composites. $\Delta C_{pn}$ represents the heat-capacity jump at T_g_ of SBR/silica composites normalized to the polymer fraction. *w* is the weight fraction of the filler. $\Delta C_{p0}$ refers to the heat-capacity jump at T_g_ of the unfilled polymer matrix, and the ∆*C*_*p*0_ of the neat SBR was 0.416 J·g^−1^·K^−1^. $\chi_{im}$ is the weight fraction of the immobilized polymer layer.

## 3. Results and Discussion

### 3.1. Aliphatic Ether Polysulfide Supported on a Silica Nanoparticle Surface

**Figure 2:**
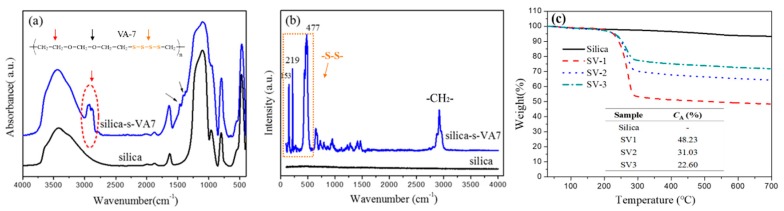
(**a**) Fourier transform infrared (FTIR) spectra, (**b**) Raman spectra of silica and silica-s-VA7, and (**c**) thermogravimetric analysis (TGA) curves of silica, SV1, SV2, and SV3.

Figure 2a shows the FTIR spectrum of pristine silica and silica-s-VA7. In the pristine-silica spectrum, characteristic absorption peaks at 1100 and 968 cm^−1^ are due to the stretching vibration of silicon hydroxyl on the silica surface, and 1630 cm^−1^ is according to the bending vibration of the adsorbed water. In the case of silica-s-VA7, two additional absorption peaks at 2930 and 2880 cm^−1^, corresponding to the stretching vibration of –CH_2_– in VA-7, are observed, and the absorption peak at 968 cm^−1^ related to Si–OH nearly disappears [1]. In addition, the O–H vibration peak at 1630 cm^−1^ also shifts to 1640 cm^−1^, indicating that the chemical environment of Si–OH has changed due to the formation of the hydrogen bond. Since the FTIR spectrum has no obvious response to the –S–S– bond, the surface of silica and silica-s-VA7 was analyzed in the Raman spectrum to further verify the surface structure of silica-s-VA7, as shown in Figure 2b. The Raman silica spectrum is a straight line without characteristic absorption peaks. The spectrum of silica-s-VA7 shows obvious vibration peaks at 153, 219, and 477 cm^−1^, which are attributed to the –S– stretching vibration peak [13]. On the other hand, a characteristic absorption peak of the methylene group appeared at 2918 cm^−1^, which was derived from the methylene group on the VA-7 molecular chain. Raman spectroscopy further demonstrated that the silica surface was successfully loaded with VA-7 to form a tetrasulfide-based supported rubber-vulcanizing agent. TGA measurement was performed to investigate the experimental loading amount of VA-7 on silica. Figure 2c shows the TGA curves of three types of silica-supported vulcanizing-agent loading with different sulfur contents. As the figure shows, from the start of the heating to around 150 °C, all samples had a mild weight-loss stage due to the evaporation of adsorbed water and dehydration of surface hydroxyl groups [19]. Significant weight loss of silica-s-VA7 concentrated in the range of 200 to 320 °C, stemming from the decomposition of the grafted organic compound, including the aliphatic ether bond and tetrasulfide bond; the weight loss of each sample gradually increased with the increase of the amount of VA7. Accordingly, the experimental loading amount of VA-7 on the silica surface of SV1, SV2, and SV3 was calculated to be 48.23%, 31.03%, and 22.60%, respectively. The high loading amount further confirmed that VA-7 was successfully grafted onto the silica surface to obtain a high sulfur-loaded vulcanizing agent. Moreover, it is worth noting that the calculated experimental loading amount for each novel vulcanizing agent could not represent the veritable sulfur content due to the aliphatic ether-bond structure of VA-7.

In order to accurately measure sulfur content in silica-s-VA7, the elemental analysis test was applied to determine the actual sulfur content on the silica-s-VA7 surface, and the results are shown in Table 2. It is obvious to note that the detected relative concentration of sulfur element of SV3, SV2, and SV1 drastically increased from 0 to 26.85% compared to that of neat silica. Moreover, the calculated sulfur content in each NR/silica-s-VA7 nanocomposite was 2.15, 2.10, and 2.13 phr per 100 phr NR in SBR/SV1, SBR/SV2, and SBR/SV3, respectively. From another perspective, the detected relative concentration of a carbon element of silica-s-VA7 obviously increased with the increase of VA-7 on the silica surface, derived from methylene on the aliphatic ether polysulfide molecular chain.

### 3.2. Vulcanization Characteristics of SBR/Silica Nanocomposites

**Figure 3:**
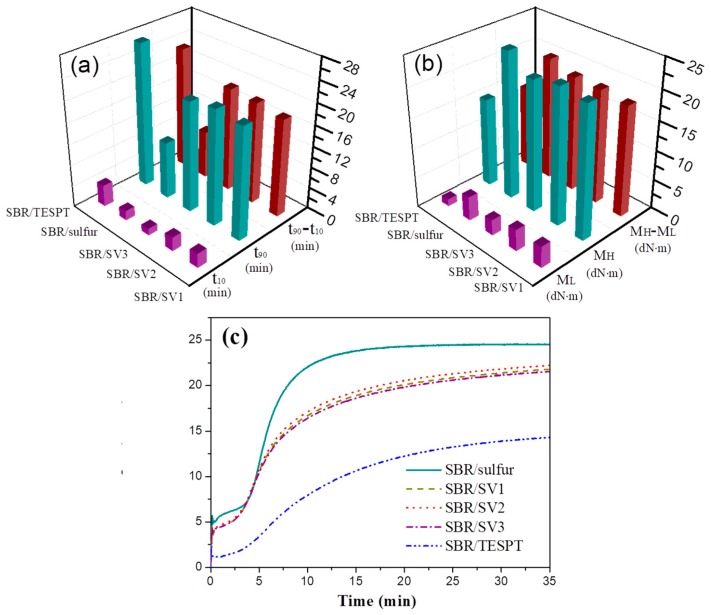
Curing parameters of SBR/sulfur, SBR/TESPT, and SBR/silica-s-VA7 nanocomposites: (**a**) time at 10% of curing degree (t_10_), optimum cure time (t_90_), and t_90_-t_10_; (**b**) minimum torque (M_L_), maximum torque (M_H_), and M_H_-M_L_; and (**c**) curing curves of SBR/silica nanocomposites.

The effect of different VA-7 contents grafted on the silica surface on the vulcanization characteristics of SBR/silica nanocomposites has been analyzed, and the curing parameters and curves of SBR/silica nanocomposites are represented in Figure 3. As shown in Figure 3a, the SBR/TESPT and SBR/silica-s-VA7 vulcanization systems exhibited higher t_90_ values and a larger difference between t_90_ and t_10_ than that of the SBR/sulfur vulcanization system, connoting the increasing vulcanizing time and lower vulcanization effect owing to the slow release of active sulfur of VA-7 and TESPT during the vulcanization process. Apart from this, the silica-s-VA7 vulcanization systems showed lower M_H_ and M_H_-M_L_ values than those of the sulfur vulcanization system, shown in Figure 3b, due to the flexibility of vulcanized rubber molecular chains originating in the aliphatic ether molecular chain of VA-7. Furthermore, the SBR/TESPT compound showed the lowest values of M_L_, M_H_, and M_H_-M_L_, probably due to insufficient crosslinking during vulcanization, and the TESPT could only serve as the interfacial compatibilizer between filler and rubber, but could not work as an effective vulcanizator. From the vulcanization curves shown in Figure 3c, the curing curves of the silica-s-VA7 vulcanization system exhibited typical vulcanization behavior. With the increase of VA-7 loading on the silica surface, the impact on the vulcanization reaction is not obvious. The vulcanization curves of SBR/silica-s-VA7 basically overlapped and showed a slow-climb tendency with higher optimum cure-time value [20].

### 3.3. Interfacial Interaction Analysis between Filler and Rubber Matrix

**Figure 4:**
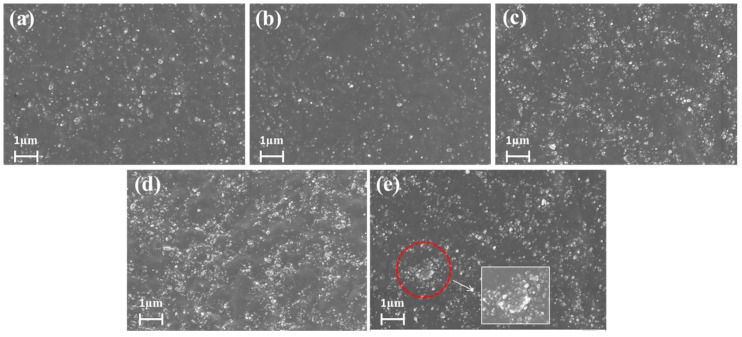
Scanning electron microscopy (SEM) images of SBR/silica nanocomposites: (**a**) SBR/SV1, (**b**) SBR/SV2, (**c**) SBR/SV3, (**d**) SBR/TESPT, and (**e**) SBR/sulfur.

The dispersion status of silica and silica-s-VA7 in the brittle section of SBR/silica nanocomposites was evaluated by SEM measurements, as shown in Figure 4. It was obvious that silica and silica-s-VA7 particles were fine-dispersed throughout the rubber matrix without visible aggregations, as shown in Figure 4a–c. According to the principle of “similar compatibility”, for one, the silica-s-VA7 carrier and the filler silica in the rubber matrix were the same inorganic phase; for another, the aliphatic ether polysulfide as the organic phase supported on the silica surface could effectively improve interfacial interaction between the filler and rubber matrix [21]. In addition, during the vulcanization process, silica-s-VA7 particles formed the reactive crosslinking points between silica-s-VA7 and the rubber molecular chains that prevent the thermodynamically favorable reaggregation of silica and silica-s-VA7. As shown in Figure 4d, TESPT as a vulcanizing agent showed better filler dispersion than that of sulfur but inferior filler dispersion than that of SBR/silica-s-VA7 composites, because TESPT works as the filler modifier to improve filler–rubber interfacial interaction. Generally, the pristine-silica surface bears numerous silanols. that tend to agglomerate and exhibit terrible dispersion in a nonpolar polymer matrix [22]. Herein, the silica surface without modification is prone to aggregate, as shown in Figure 4e.

**Figure 5:**
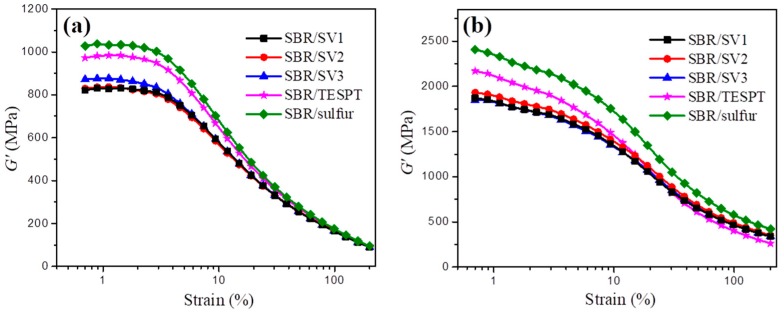
Dependence of *G′* of (**a**) unvulcanizated (**b**) vulcanizated SBR/silica nanocomposites.

The dependence of shear modulus (*G′*) and of the uncured and elastic modulus (*G′*) of the cured SBR/silica-s-VA7, SBR/TESPT, and SBR/sulfur nanocomposites on the strain obtained through the rubber processibility analyzer (RPA) are shown in Figure 5. For the uncured nanocomposites shown in Figure 5a, the shear modulus *G′* of different vulcanization systems exhibits a nonlinear relationship with increasing strain. As the strain increases (strain > 3%), the *G′* of SBR/silica nanocomposites shows a tendency to sharply decline, which is called the Payne effect. Due to the surface modification of silica, the value of *G′* for SBR/silica-s-VA7 and SBR/TESPT nanocomposites is considerably decreased and lower than that of the SBR/sulfur nanocomposite, denoting that VA-7 and TESPT are helpful in weakening the interactions between silica and the Payne effects. The mitigated network of silica-s-VA7 and TESPT can be attributed to the weakened interactions among silica particles, resulting from the decrease of hydrogen bonds between the abundant hydroxyl groups and absorbed water on the particle surface.

The dependence of the elastic modulus (*G′*) of the vulcanized SBR/sulfur, SBR/TESPT, and SBR/silica-s-VA7 nanocomposites on the strain is shown in Figure 5b. The SBR/sulfur nanocomposite has been revealed to have higher value of initial *G′* than that of the SBR/silica-s-VA7 and SBR/TESPT nanocomposites, with equal filler content and a more obvious Payne effect. However, the influence of the different VA-7-supporting ratios on the silica surface in the SBR/silica-s-VA7 nanocomposites on the *G′* value is not obvious; the flexibility of the molecular chain rooted in the aliphatic ether-chain segment comes to a lower *G′* value than that of SBR/sulfur with increasing strain. In general, the silica-s-VA7 vulcanization system can effectively decrease the Payne effect of the nanocomposites and improve the filler–rubber interfacial interaction.

### 3.4. Immobilized Polymer Layer Anchoring on Filler Surface

**Figure 6:**
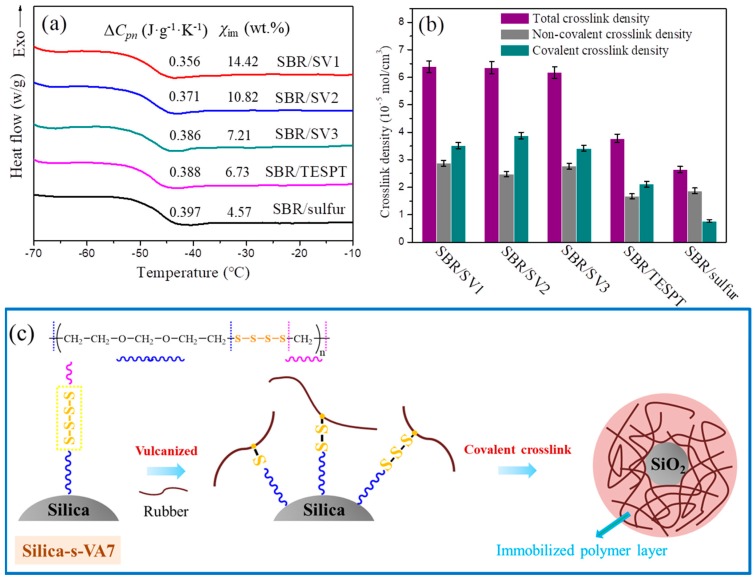
(**a**) DSC curves of SBR/silica nanocomposites at the glass-transition region, (**b**) crosslinking density of SBR/silica nanocmoposites, and (**c**) interfacial reaction in SBR/silica-s-VA7 composites during vulcanization.

To further investigate the filler–rubber interfacial interactions in SBR nanocomposites, the immobilized polymer layer approaching on the filler surface is calculated by DSC [12]. The immobilized polymer layer bridges the interface between filler and rubber, which can influence interfacial strength in rubber composites. Figure 6a reveals the DSC curves of filled SBR nanocomposites vulcanized by silica-s-VA7, TESPT, and sulfur, respectively, with the uniform filler content. It is a truism that Δ*C_pn_* at glass transition is closely related to the freedom of molecular motion, and the lower value of Δ*C_pn_* represents the weaker ability of molecular motion [23]. As shown in Figure 6a, the Δ*C_pn_* of SBR/silica-s-VA7 composites is lower than that of SBR/TESPT and SBR/sulfur composites, revealing that more rubber molecular chains are immobilized embracing silica-s-VA7 than those embracing silica in SBR/sulfur nanocomposites and SBR/TESPT. This is also reflected by the results of the value of the weight fraction of immobilized polymer layer $\chi_{im}$. The $\chi_{im}$ of SBR/silica-s-VA7 nanocomposites is increased with the increasing proportion of VA7 and silica, and is increased by 60%–300% compared with that of SBR/sulfur and SBR/TESPT composites, demonstrating stronger interfacial interaction between silica-s-VA7 and rubber [24,25]. It is due to that fact that silica-s-VA7 nanoparticles as a novel-vulcanizing agent generate crosslinking points on their surface during the vulcanization process, and crosslink numerous rubber molecular chains around the silica-s-VA7 surface. For SBR/TESPT composites, TESPT sulfide linkage bonded to the silica surface can be dissociated and react with SBR chain to form a crosslink between the silica and rubber. In the sulfur vulcanization process, the filler surface has not directly participated in the crosslinking process with the rubber molecular chains, and the rubber molecular chains adhere to the silica surface merely through entanglement.

Crosslinking density, shown in Figure 6b, demonstrated a similar tendency as the result of the immobilized polymer layer in SBR/silica nanocomposites. The values of total crosslink density of SBR/silica-s-VA7 nanocomposites are almost similar, but much higher than in sulfur and TESPT vulcanizing systems. Elemental sulfur can be formed from VA-7 under a curing temperature and it can participate in crosslinking reactions to increase crosslinking density. Furthermore, SBR/silica-s-VA7 nanocomposites have a higher value of covalent crosslink density than that of noncovalent crosslink density, which is contrary to the SBR/sulfur nanocomposite but similar to the SBR/TESPT nanocomposite. It is demonstrated that the novel vulcanizing agent silica-s-VA7 can significantly increase crosslinking density and improve the interfacial interaction between filler and rubber compared to that of a traditional sulfur vulcanization system.

It is well known that the vulcanizing agent is an indispensable rubber additive in the process of rubber crosslinking and it endows the rubber composite with excellent mechanical performance [26,27]. VA-7 molecules react with silica to form hydrogen bonds, and the polarity of silica surface is reduced due to the modification. The polar cure accelerator molecules are less adsorbed on the silica-s-VA7 surface than on the unmodified one, which leads to an increase in the content of the free cure accelerator. During the vulcanization process, the novel supported vulcanizing agent generates sulfur active points and forms a chemical bond between silica-s-VA7 and rubber chains. As shown in Figure 6c, VA-7 supported on the silica surface can fracture into a mono-, di-, and trisulfide bond according to the lower bond energy of –S–S– than that of –C–S– during the vulcanization process. These vulcanization points react with the *α*-H on the rubber chain and form a rubber-silica-rubber organic–inorganic crosslinking network structure. Meanwhile, the rubber chains wrap around silica-s-VA7 particles via chemical bonding to form a thicker immobilized polymer layer, improve interfacial interaction, and remarkably enhance crosslink density.

### 3.5. Mechanical Properties of SBR/Silica Nanocomposites

**Figure 7:**
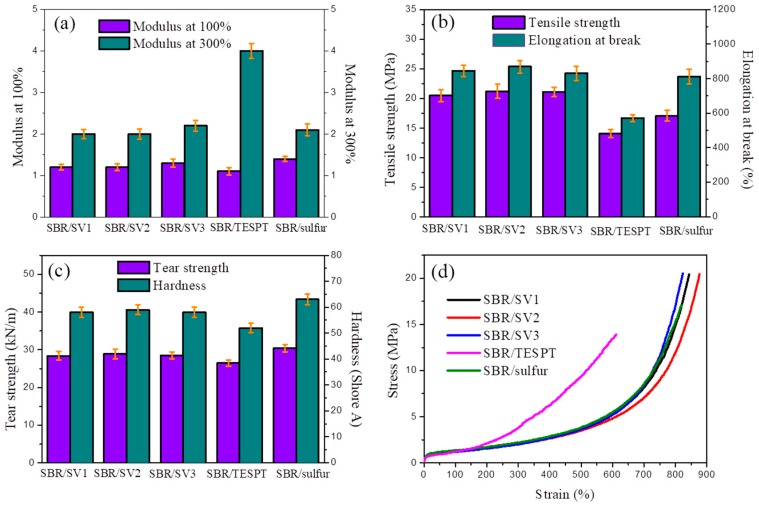
Mechanical properties of SBR/silica nanocomposites: (**a**) modulus at 100% and 300%, (**b**) tensile strength and elongation at break, (**c**) tear strength and hardness, and (**d**) stress-strain curves of SBR/silica nanocomposites.

The mechanical properties of SBR/silica nanocomposites vulcanized by different vulcanizing agents are presented in Figure 7a–c, and the representative stress–strain curves are shown in Figure 7d. It can be revealed that the novel vulcanizing agent silica-s-VA7 in the rubber vulcanization process is able to meet the practical requirements of rubber materials. For instance, the SBR/silica-s-VA7 nanocomposites achieved approximately 17.6% to 23.4% increases in tensile strength, and stretchabilities had comparable improvement, which compared with SBR/sulfur nanocomposites, even though the 100% and 300% modulus had no obvious changes. It is a fact that, during the vulcanization process, the active vulcanizing point generated from silica-s-VA7 formed the mono- and disulfide bonds to crosslink the rubber molecular chains with higher bond energy than that of the sulfur vulcanization system, which forms the dominating polysulfide bond [13,28,29]. Comparing with the SBR/TESPT system, which possesses a similar structure to tetrasulfide, SBR/silica-s-VA7 nanocomposites exhibit better mechanical properties than those of SBR/TESPT nanocomposites. SBR/silica-s-VA7 nanocomposites also achieved approximately threefold increases in crosslinking density and covalent crosslinking density on account of a mass of rubber molecular chains that were immobilized on the silica-s-VA7 and silica surface to form a thicker immobilized polymer layer. In general, the thicker immobilized polymer layer between the filler and rubber molecular chains can lead to significant improvement on the interfacial interface of filler and reinforcement of the mechanical properties of the SBR/silica-s-VA7 nanocomposites. However, the tear strength of SBR/silica-s-VA7 nanocomposites shows a mild decrease due to the fact that flexible molecular chain segments originating from aliphatic ethers exhibited a plasticizing effect, which affected the hardness of the SBR/silica-s-VA7 nanocomposites. In general, the supporting rubber additives, such as the vulcanizing agent, accelerator and antioxidant, onto the silica surface can lead to reinforcement of the mechanical properties of rubber materials [30,31,32], and usually bring in several advantages, such as overcoming the easy volatility, migration, and dissolution of small molecular additives.

## 4. Conclusions

In this article, we reported a novel organic–inorganic hybrid vulcanizator instead of sulfur to obtain a surface-functionalized nanofiller (silica-s-VA7) for rubber vulcanization and improving filler dispersion. Silica-s-VA7 could be uniformly dispersed into an elastomer with further enhanced filler–rubber interaction compared with traditional sulfur and TESPT vulcanization systems. The supported vulcanizing agent silica-s-VA7 served as a chemical crosslinker, which gave rise to primary covalent network-interlinked rubber chains and filler, and significantly improved the immobilized polymer layer and the crosslinking density for SBR/silica-s-VA7 nanocomposites. Moreover, SBR/silica-s-VA7 nanocomposites exhibited greater mechanical strength than SBR/sulfur and SBR/TESPT composites, which may be a valuable insight for reinforcing rubber composites and provide a new avenue to designing functionalized nanoparticles in the rubber industry.

## Figures and Tables

**Table 1:** Formulas of styrene-butadiene rubber SBR1502 (SBR)/silica nanocomposites. Note: SV1, 10 g silica vs. 10 g VA-7; SV2, 20 g silica vs. 10 g VA-7; SV3, 30 g silica vs. 10 g VA-7; TESPT, tetrasulfide; CBS, n-cyclohexyl-2-benzothiazolesulfenamide; ZNO, zinc oxide; SA, stearic acid; DMPPD, n-1,3-dimethylbutyl-N’-phenyl-p-phenylenediamine.

Sample	Component (phr)										
	SBR	SV1	SV2	SV3	Silica	S	CBS	ZnO	SA	TESPT	DMPPD
**SBR/SV1**	100	8	0	0	32	0	2	4	2	0	2
**SBR/SV2**	100	0	12	0	28	0	2	4	2	0	2
**SBR/SV3**	100	0	0	16	24	0	2	4	2	0	2
**SBR/TESPT**	100	0	0	0	38	0	2	4	2	8	2
**SBR/sulfur**	100	0	0	0	38	2	2	4	2	0	2

**Table 2:** Elemental analysis of silica and silica-s-VA7.

	Content (%)		
	C	H	S
**Silica**	0	1.56	0
**SV1**	13.15	2.21	26.85
**SV2**	8.37	1.60	17.52
**SV3**	6.55	1.30	13.30
